# Neural Dynamics of Strategic Early Predictive Saccade Behavior in Target Arrival Estimation

**DOI:** 10.3390/brainsci15070750

**Published:** 2025-07-15

**Authors:** Ryo Koshizawa, Kazuma Oki, Masaki Takayose

**Affiliations:** 1 College of Economics, Nihon University, Tokyo 101-0061, Japan; 2 College of Science and Technology, Nihon University, Chiba 274-8501, Japan; oki.kazuma@nihon-u.ac.jp; 3 College of Industrial Technology, Nihon University, Chiba 275-8576, Japan; takayose.masaki@nihon-u.ac.jp

**Keywords:** EEG, low beta oscillations, frontal eye field, middle temporal visual area, predictive saccades

## Abstract

**Background/Objectives**: Accurately predicting the arrival position of a moving target is essential in sports and daily life. While predictive saccades are known to enhance performance, the neural mechanisms underlying the timing of these strategies remain unclear. This study investigated how the timing of saccadic strategies—executed early versus late—affects cortical activity patterns, as measured by electroencephalography (EEG). **Methods**: Sixteen participants performed a task requiring them to predict the arrival position and timing of a parabolically moving target that became occluded midway through its trajectory. Based on eye movement behavior, participants were classified into an Early Saccade Strategy Group (SSG) or a Late SSG. EEG signals were analyzed in the low beta band (13–15 Hz) using the Hilbert transform. Group differences in eye movements and EEG activity were statistically assessed. **Results**: No significant group differences were observed in final position or response timing errors. However, time-series analysis showed that the Early SSG achieved earlier and more accurate eye positioning. EEG results revealed greater low beta activity in the Early SSG at electrode sites FC6 and P8, corresponding to the frontal eye field (FEF) and middle temporal (MT) visual area, respectively. **Conclusions**: Early execution of predictive saccades was associated with enhanced cortical activity in visuomotor and motion-sensitive regions. These findings suggest that early engagement of saccadic strategies supports more efficient visuospatial processing, with potential applications in dynamic physical tasks and digitally mediated performance domains such as eSports.

## 1. Introduction

The ability to accurately predict the arrival position of a moving target is fundamental to sports performance, motor control, and everyday activities. Such prediction requires the brain to process dynamic visual information, including the target’s velocity and direction [1]. For instance, neurons in the middle temporal (MT) visual area integrate signals from motion-sensitive cells in the primary visual cortex to detect and interpret object motion [2,3,4,5]. A key way the brain processes visual motion is by guiding the image of a moving target onto the fovea—the central, high-acuity region of the retina—through a combination of smooth pursuit or saccadic eye movements [6]. Optimizing visual tracking typically involves coordination between both systems. This coordination relies on shared sensory inputs such as position and velocity errors, which are evaluated to determine whether correction should be executed through smooth pursuit alone or supplemented by catch-up saccades [6]. Thus, smooth pursuit and saccadic systems operate in tandem to maintain accurate target tracking. However, this coordination presuppose continuous visual access to the moving target. In contrast, the neural mechanism supporting early prediction of arrival position—prior to or independent of continuous tracking—remains insufficiently understood.

A commonly used paradigm to assess the predictability of the arrival position in a controlled setting involves occluding the target midway through its trajectory [7,8]. Koshizawa et al. [9] employed a similar design, requiring participants to predict the arrival location of a partially occluded moving target. Unlike prior studies, their task explicitly manipulated prediction strategies and examined their neural correlates using electroencephalography (EEG). Specifically, they used EEG to investigate cortical activity associated with a saccadic strategy, in which participants shifted their gaze early to the anticipated arrival location, rather than continuously tracking the target in the pursuit strategy task. Moreover, they compared neural activity between participants who successfully predicted the arrival location using the saccade strategy and those who did not. This comparison enabled assessment of whether accurate prediction could be achieved within a restricted temporal window. Their findings showed that participants who employed the saccade strategy exhibited increased EEG activity at electrodes over the visual, parietal, and motor cortices (Oz, CP2, Cz), indicating engagement of visuospatial attention and predictive mechanisms. Notably, participants who successfully predicted the arrival location also showed elevated EEG activity at the P7 electrode, suggesting enhanced processing of target speed and direction in the motion-sensitive MT area.

However, previous research has not examined how the *timing* of saccade execution affects neural activity. Given the limited window available for prediction, the effectiveness of a saccadic strategy may critically depend on whether it is implemented early. A delayed saccade might negate its strategic advantage. Although predictive saccade behavior has been explored in various contexts, little is known about how the temporal characteristics of such strategies relate to underlying neural activity during predictive visuomotor tasks. To address this gap, the present study aimed to elucidate the neural dynamics associated with the timing of saccadic strategy execution during the prediction of a target’s arrival location.

These findings have practical implications for high-speed dynamic tasks such as baseball batting, where rapid prediction is required within a very short time window. They may also be relevant to digital domains such as eSports. Although traditional ball sports and eSports differ in physicality, they demand rapid visual processing and decision-making under time constraints. In such contexts, deliberately shifting the gaze away from the target can improve performance. For example, in first-person shooter (FPS) games, expert players often avoid fixating on a single opponent. Instead, they employ rapid saccades to scan the environment, briefly diverting their gaze to assess surrounding threats. These dynamic gaze shifts reflect a sophisticated visual strategy shaped by task demands. By examining the neural underpinnings of such strategies, the present study may contribute to enhancing performance in traditional athletic training and eSports.

## 2. Materials and Methods

The experimental design was based on the paradigm developed by Koshizawa et al. [9], which investigated EEG activity associated with pursuit and saccadic visual strategies. However, the present study introduced a key modification: the novel classification of participants into an Early Saccade Strategy Group (SSG) and a Late SSG based on the timing of their eye movements. This classification enabled a more nuanced investigation into the neural dynamics underlying predictive saccade strategies.

### 2.1. Participants

Sixteen healthy adults (mean age: 20.13 years; 14 men) participated in this study. Only participants whose eye movements could be accurately recorded using the Gazefinder^®^ infrared eye-tracking system (JVC Kenwood, Yokohama, Japan) were included in the analysis. All participants had normal or corrected-to-normal vision and reported no history of psychological, psychiatric, or neurological disorders.

Written informed consent was obtained from all participants prior to this study. The research protocol was approved by the Ethics Review Committee on Research with Human Subjects at the College of Industrial Technology, Nihon University (Approval No. S2020–008), and adhered to the principles outlined in the Declaration of Helsinki. Participants were instructed to abstain from alcohol and drug use in the days leading up to the experiment to minimize potential confounding effects.

### 2.2. Apparatus

#### 2.2.1. Eye Position Acquisition

Eye-tracking data were recorded using the Gazefinder^®^ infrared eye-tracking system (JVC Kenwood). Stimuli were presented on a 19-inch monitor (1280 × 1024 pixels) equipped with built-in infrared cameras. The monitor was positioned approximately 60 cm from the participants’ eyes and subtended 35.662° horizontally and 28.862° vertically within the visual field. The X and Y coordinates of gaze position were sampled at 50 Hz. Prior to each experimental session, a 5-point calibration procedure was conducted to ensure accurate eye position tracking.

#### 2.2.2. Electroencephalography

EEG data were collected using the eego™ sports system (ANT Neuro, Hengelo, The Netherlands) with 32 Ag/AgCl electrodes positioned according to the international 10–20 system. Signals were recorded from the following electrodes: Fz, F3, F4, FC1, FC2, FC5, FC6, Cz, C3, C4, T7, T8, CP1, CP2, CP5, CP6, Pz, P3, P4, P7, P8, POz, Oz, O1, and O2. Electrodes Fpz, Fp1, Fp2, F7, and F8 were excluded from analysis a priori, based on previous studies [9,10,11], due to their susceptibility to ocular artifacts. To synchronize EEG recordings with stimulus onset, a photosensor (Miyuki Giken, Tokyo, Japan) was attached to the monitor. EEG signals were referenced to CPz, with AFz serving as the ground. Data were sampled at 1000 Hz, and electrode impedance was maintained below 20 kΩ [12]. Prior to analysis, EEG data were re-referenced to the common average reference. While the overall setup was consistent with previous studies on EEG and visual prediction [9,10,11], this study specifically focused on comparing neural activity between participants in the Early and Late SSGs.

### 2.3. Task

The experimental paradigm was adapted from Koshizawa et al. [9], who examined EEG activity associated with pursuit and saccadic visual strategies. The present study, however, introduces a novel classification of participants into Early and Late SSGs and focuses specifically on the temporal dynamics of EEG signals associated with predictive saccade execution.

**Figure 1:**
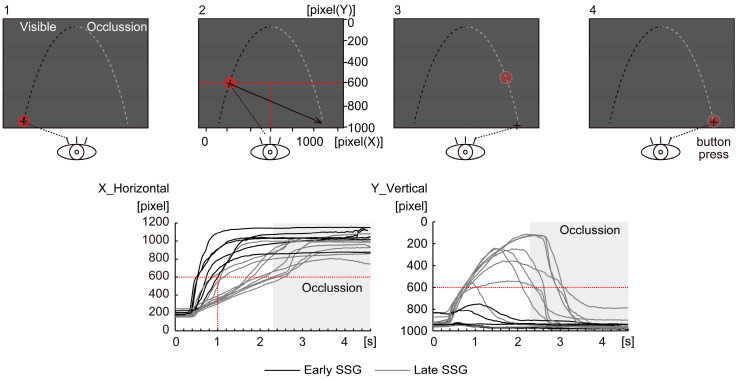
Experimental design and eye position profiles. Participants were instructed to predict the final landing point of a target moving along a parabolic trajectory from the lower left to the lower right of the screen. The target was visible during the first half of its trajectory and occluded during the second half (1). Upon estimating the arrival position—approximately aligned with the initial vertical level—participants were required to execute a saccade to the predicted location (2), maintain their gaze at that position (3), and press a button with their right thumb when they believed the target had arrived (4). The bottom panels display representative eye position trajectories for all participants, categorized into the Early Saccade Strategy Group (Early SSG, black lines, *n* = 6) and the Late Saccade Strategy Group (Late SSG, gray lines, *n* = 10). The left graph shows horizontal (*X*-axis) eye positions, while the right graph illustrates vertical (*Y*-axis) eye positions over time. Red dotted lines indicate the classification thresholds: 600 pixels for both *X*- and *Y*-coordinates and 1 s after target onset for the temporal criterion (applied to both axes). Participants were classified into the Early SSG if their gaze rapidly shifted horizontally (*X* ≥ 600 pixels within 1 s) and remained fixated below the *Y*-threshold (*Y* ≥ 600 pixels) within the same 1 s window. These thresholds were derived from previous research [9], which reported similar gaze behavior in a saccade strategy task. In that study, participants typically reached approximately 600 pixels in horizontal eye position within 1 s of target onset, and vertical gaze position consistently remained in the lower portion of the display. These thresholds were applied to define early predictive saccade behavior in a reproducible and objective manner. The shaded gray region represents the occlusion period (after 2.3 s), during which the target was no longer visible. The task was designed to simulate gravitational acceleration and included three trajectory variations (CLOSE, MID, and DISTANT), which systematically manipulated initial velocity, launch angle, and final position under the assumption of no air resistance.

As illustrated in Figure 1, the task was designed to investigate the predictive mechanisms underlying saccadic strategies when a moving target becomes occluded. Participants were instructed to predict the final arrival position and arrival time of a parabolically moving target that traveled from the lower left to the lower right of the screen. The target’s trajectory simulated motion under gravitational acceleration, with the final landing position located at the same vertical level as the launch point. To prevent learning effects and response habituation, three variations of the target trajectory—CLOSE, MID, and DISTANT—were randomly presented across trials. These variations were achieved by systematically manipulating the target’s initial velocity, launch angle, and horizontal distance, while assuming motion in a vacuum (i.e., no air resistance). This task structure enabled the examination of neural responses under varying predictive demands and allowed for the classification of participants based on the timing of their saccadic responses relative to target occlusion.

#### 2.3.1. Target Characteristics

The moving target was a red circle with a radius of 0.38° of visual angle, displayed against a gray background. Each target followed a parabolic trajectory for a total duration of 4.6 s. The first 2.3 s of motion were visible to the participants, while the remaining 2.3 s were occluded by a gray mask. Target trajectories varied across three conditions (CLOSE, MID, and DISTANT) based on differences in launch angles and initial velocities, as summarized in Table 1.

To simulate naturalistic motion dynamics (within the screen space), the horizontal and vertical components of the target’s path were modeled using standard kinematic equations under uniform gravitational acceleration of 9.81 cm/s^2^. Specifically, the target’s position at each time *t* was computed as follows: (1) horizontal position: *x* = *v*_0_·cos(*θ*)·*t*; (2) vertical position: *y* = *v*_0_·sin(*θ*)·*t* − ½·*g*·*t*^2^; (3) where *v*_0_ is the initial velocity, *θ* is the launch angle, and *g,* the gravitational acceleration, is set at 9.81 cm/s^2^ to approximate Earth gravity within the screen’s scaled environment.

#### 2.3.2. Task Procedure

Participants completed a saccade strategy task designed to assess their ability to execute predictive saccades (as illustrated in Figure 1). Each trial consisted of the following phases: (1) Fixation phase: a stationary target (red circle) appeared at the starting position for a randomized duration of 2.4–3.6 s. (2) Motion phase: The target moved along a parabolic path for 4.6 s. The first half (2.3 s) was fully visible; the second half (2.3 s) was occluded. (3) Response phase: participants were instructed to shift their gaze to the predicted final arrival location of the target as soon as they made their prediction, maintain fixation at the predicted location until they estimated that the target had arrived, and press a response button with their right thumb at the moment they judged the target had reached its final position/endpoint.

Prior to the main trials, participants completed a practice session involving a mirrored version of the task (right-to-left motion). The main experiment comprised six blocks of 30 randomized trials each, with a 2 min rest period between blocks. Trial order was randomized.

### 2.4. Data Analysis

Trials were excluded from further analysis based on the following criteria: (1) loss of eye position data due to blinking during the presentation of the moving target; (2) signal loss or tracking failure by the eye-tracking system; (3) EEG signal deflections exceeding ±100 μV in any channel during the time window from 200 ms before to 2300 ms after target onset, following artifact rejection procedures established in prior research [12]. Additionally, EEG data corresponding to the occluded segment of the target’s trajectory (after 2300 ms) were excluded from analysis, as this phase was expected to involve increased eye movement and a higher likelihood of artifacts. However, eye-tracking data were retained for the full duration of the target interval (0–4600 ms).

Although the target trajectories varied in endpoint distance (CLOSE, MID, and DISTANT), they were included primarily to prevent response habituation by introducing trial-by-trial variability in target motion. Since the aim of the present study was to investigate the temporal dynamics of predictive saccade strategies—rather than the effects of trajectory length or endpoint location—all trajectory conditions were collapsed during data analysis. This approach ensured a sufficient number of artifact-free trials per participant and enhanced the robustness of time-locked neural measures.

To classify participants into distinct saccade strategy groups, eye position data were closely examined with respect to horizontal (*X*-axis) and vertical (*Y*-axis) gaze behavior. As illustrated in Figure 1, classification was based on gaze patterns within the first second following target onset. Threshold values (600 pixels for both *X-* and *Y*-axes and 1 s for timing) were derived from previous research [9], which employed a similar predictive saccade task. In that study, participants in the saccade strategy task typically reached approximately 600 pixels in the horizontal direction within 1 s after target onset. The same threshold was adopted here for consistency and reproducibility. For the *Y*-axis, gaze in the previous study [9] consistently remained directed toward the lower part of the display, exceeding 600 pixels. In the current coordinate system, *Y*-values increase in the downward direction. Although that study did not specify a temporal window for the *Y*-axis, we applied a consistent 1 s window to parallel the horizontal criterion and ensure methodological clarity. Participants whose gaze met both criteria—horizontally reaching 600 pixels within 1 s and vertically fixating in the lower portion of the screen (*Y* ≥ 600 pixels)—were classified into the Early Saccade Strategy Group (Early SSG, *n* = 6), characterized by early predictive saccades toward the anticipated landing point. Participants who either delayed their saccadic response or continued to visually track the target along its trajectory were classified into the Late SSG (*n* = 10). Although classification was based on threshold criteria of both axes, time-series plots of horizontal and vertical eye positions (*X* and *Y* positions) were generated for all participants to visually confirm group assignment (see Figure 1). EEG data from all artifact-free trials were included in the subsequent analysis, regardless of the target condition (CLOSE, MID, or DISTANT), allowing for a unified evaluation of the neural dynamics associated with predictive saccade behavior across participants.

#### 2.4.1. Eye Position

The primary behavioral index used to evaluate participants’ ability to predict the final arrival position of the moving target was position error (PE). PE was defined as the Euclidean distance between the participant’s predicted landing position—indicated by their gaze location at the time of response—and the actual final position of the target at the bottom of the display. This metric, referred to as PE at response, reflects spatial accuracy in predicting the target’s trajectory endpoint.

An additional behavioral measure, response error, was computed by subtracting the actual target arrival time (4.6 s) from the participant’s response time (i.e., the interval from target motion onset to the button press with the right thumb). This index served as a temporal measure of predictive accuracy.

To assess the evolution of prediction accuracy over time, time-resolved PE was calculated throughout the 4.6 s trajectory of the target. Specifically, at each sampling point (50 Hz; every 20 ms), the Euclidean distance between the participant’s gaze position and the final target location was computed and stored. This produced a continuous time-series of PE values from 0.02 to 4.60 s (i.e., PE at point), capturing the dynamic process of spatial prediction.

In addition, raw eye position coordinates (*X* and *Y* values) at each time point were recorded as a time-series. These data were analyzed to characterize gaze behavior across the entire target trajectory and to elucidate the temporal dynamics of visual strategies employed by participants. Together, these metrics enabled a comprehensive evaluation of how eye movements evolved relative to the predicted final position of the target.

#### 2.4.2. Electroencephalography

EEG was employed to investigate the neural correlates underlying predictive saccade strategies. Specifically, neural oscillatory activity in the low beta frequency band (13–15 Hz)—a range associated with visuomotor integration, attention, and motor preparation—was analyzed using the Hilbert transform [13] to compute the instantaneous log amplitude of the complex analytic signal. EEG data processing was conducted using EMSE Suite v5.6 (Cortech Solutions, Inc., Wilmington, NC, USA). To enhance signal fidelity, a rectifier filter was applied prior to log_10_ transformation and baseline correction via polynomial detrending. These preprocessing steps ensured that amplitude signals oscillated symmetrically around zero, preserving the structure of the underlying EEG while enabling improved comparisons across participants and conditions.

Group-level comparisons were conducted by computing grand-averaged waveforms of the log-transformed low-beta-band amplitudes, following conventional event-related potential (ERP) analysis procedures. These waveforms were used to examine differences in low beta activity between the Early SSG and Late SSG participant groups. Furthermore, topographic scalp maps were generated by averaging amplitudes across participants within time windows that exhibited statistically significant between-group differences. This allowed for the localization of cortical regions implicated in predictive saccade execution, particularly during periods of early anticipation of target arrival.

While definitions of the low beta range vary across the literature (e.g., 8–15 Hz, 12–24 Hz, 13–30 Hz), the 13–15 Hz band chosen in this study aligns with prior work identifying this range as most relevant to attentional modulation and visuomotor processes [14,15,16].

### 2.5. Statistical Analysis

#### 2.5.1. Eye Position

Although the false discovery rate (FDR) correction was applied to control for Type I error inflation, unadjusted *p*-values are reported for transparency, in line with current statistical reporting guidelines. This ensures that the reported differences reflect true effects rather than artifacts of multiple comparisons. To evaluate differences in predictive gaze behavior between the two saccade strategy groups, statistical analyses were conducted on four key eye movement parameters: (1) PE at response—the Euclidean distance between the predicted and actual target landing positions at the time of the button press; (2) response error—the temporal deviation from the target’s expected arrival time (4.6 s), calculated as the participant’s response time minus 4.6 s; (3) PE over time (0.02–4.60 s)—a time-series of gaze error relative to the final arrival position throughout the target’s 4.6 s trajectory; (4) eye position (XY coordinates, 0.02–4.60 s)—the recorded spatial location of the gaze over time.

Participants were classified into two groups: Early SSG (*n* = 6) and the Late SSG (*n* = 10). For PE at response and response error (parameters 1 and 2), independent (unpaired) *t*-tests were conducted to compare group performance. For the time-series measures (parameters 3 and 4), statistical comparisons involved multiple time points, and thus the Benjamini–Hochberg FDR correction was applied. Importantly, all values that were statistically significant before correction remained significant after FDR adjustment; however, in line with recommended practices, only unadjusted *p*-values are reported here.

All statistical analyses were performed using IBM SPSS Statistics 26, with the significance threshold set at *p* ≤ 0.05.

#### 2.5.2. Electroencephalography

To investigate neural differences between the Early and Late SSGs, statistical comparisons were conducted on dynamic changes in low beta EEG activity (13–15 Hz). Given the event-related nature of the task and the continuous EEG time-series, non-parametric permutation tests were used to compare groups at each 1 ms time point across the analysis window and 25 EEG channels, following established guidelines for time-series EEG analysis [17,18]. These tests were implemented in EMSE Suite v5.6 (Cortech Solutions, Inc.), with the Hilbert transform applied to extract the instantaneous log amplitude of the low beta band, as described in previous studies [13]. To address the issue of multiple comparisons and spatial correlation among electrodes, a spatial cluster-based correction method was employed, leveraging a permutation-based approach that accounts for topographical dependencies. Following prior literature [9,10], an effect was considered statistically significant if (1) the computed *p*-value was ≤0.05 and (2) the effect was sustained for at least 10 ms, reducing the risk of detecting transient or spurious fluctuations [19]. Additionally, topographic scalp maps were created to visualize the spatial distribution of significant group differences. For electrodes showing significant effects across adjacent time windows, the maps were generated by averaging low beta power across those intervals.

Unlike prior studies that focused on average EEG power during pursuit or saccade tasks [9], the current analysis emphasizes the temporal evolution of low beta activity and its relationship to predictive saccade execution. These statistical procedures provide detailed insight into the cortical dynamics associated with distinct early versus late predictive saccade strategies.

## 3. Results

### 3.1. Eye Position

**Figure 2:**
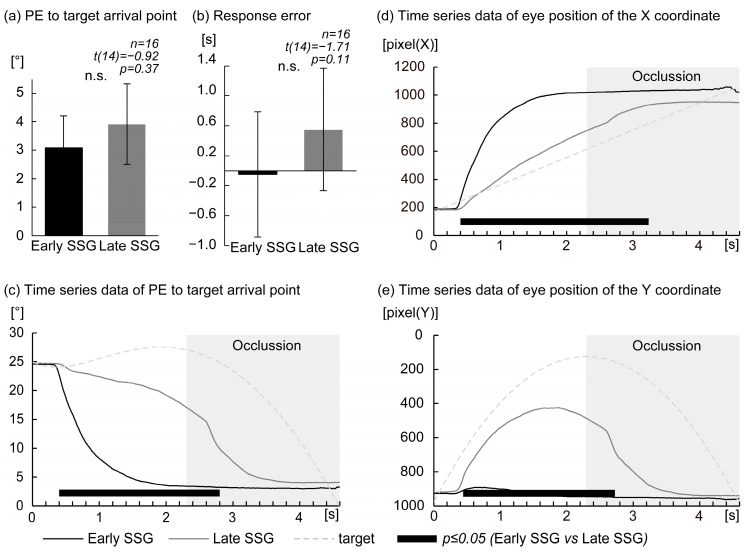
Eye movement data comparison between Early and Late Saccade Strategy Groups. (**a**) Comparison of position error (PE) at the time of response to the target’s arrival point between the Early Saccade Strategy Group (Early SSG) and the Late Saccade Strategy Group (Late SSG). (**b**) Comparison of response error between the Early SSG and Late SSG. (**c**) Time-series data of PE relative to the target’s arrival point; black and gray lines represent the Early and Late SSG, respectively. The dashed trace indicates the trajectory of the moving target. (**d**) Time-series data of horizontal (*X*-axis) eye position; black and gray lines represent the Early and Late SSGs, respectively, with the dashed trace representing the moving target. (**e**) Time-series data of vertical (*Y*-axis) eye position, with the same representation as in (**d**). In panels (**a**,**b**), “n.s.” indicates non-significant differences. Error bars represent ±1 standard error of the mean (SEM). In panels (**c**–**e**), the *X*-axis indicates time from target onset. Black segments beneath each graph indicate intervals with significant between-group differences (*p* ≤ 0.05, *t*-test).

The comparison of PE at the response point between the Early SSG and Late SSGs revealed no significant difference (Early SSG: 3.08 ± 1.13°, Late SSG: 3.92 ± 1.41°; *t*(14) = −0.92, *p* = 0.37) (Figure 2a).

Similarly, no significant difference was observed in response error between the groups (Early SSG: −0.05 ± 0.83°, Late SSG: 0.55 ± 0.82°; *t*(14) = −1.71, *p* = 0.11) (Figure 2b).

Time-series analysis of PE to the final target position is illustrated in Figure 2c. Compared to the Late SSG, the Early SSG showed significantly reduced PE during specific time intervals following target onset: 0.40–2.80 s (*p* ≤ 0.05 at 0.40–0.44 s, 2.58–2.80 s; *p* < 0.01 at 0.46–0.62 s, 1.96–2.56 s; *p* < 0.001 at 0.64–1.94 s).

Figure 2d presents the time-series for horizontal (*X*-axis) eye position. The Early SSG exhibited significantly greater *X*-axis gaze positions than the Late SSG during the interval from 0.40–3.22 s after target onset (*p* ≤ 0.05 at 0.40–0.46 s, 2.64–3.22 s; *p* < 0.01 at 0.48–0.70 s, 2.04–2.62 s; *p* < 0.001 at 0.72–2.02 s).

Similarly, time-series data for the vertical (*Y*-axis) eye position are shown in Figure 2e. The Early SSG demonstrated significantly higher *Y*-axis gaze positions compared to the Late SSG across 0.44–2.74 s (*p* ≤ 0.05 at 0.44–0.46 s, 2.54–2.74 s; *p* < 0.01 at 0.48–0.64 s, 1.36–2.52 s; *p* < 0.001 at 0.66–1.34 s).

### 3.2. Electroencephalography

To examine differences in EEG activity between the Early SSG and Late SSGs, non-parametric paired permutation tests were conducted at each electrode during the 2.3 s visible trajectory period. Spectral analysis focused on the low beta frequency range (13–15 Hz), with instantaneous log amplitude extracted using the Hilbert transform.

**Figure 3:**
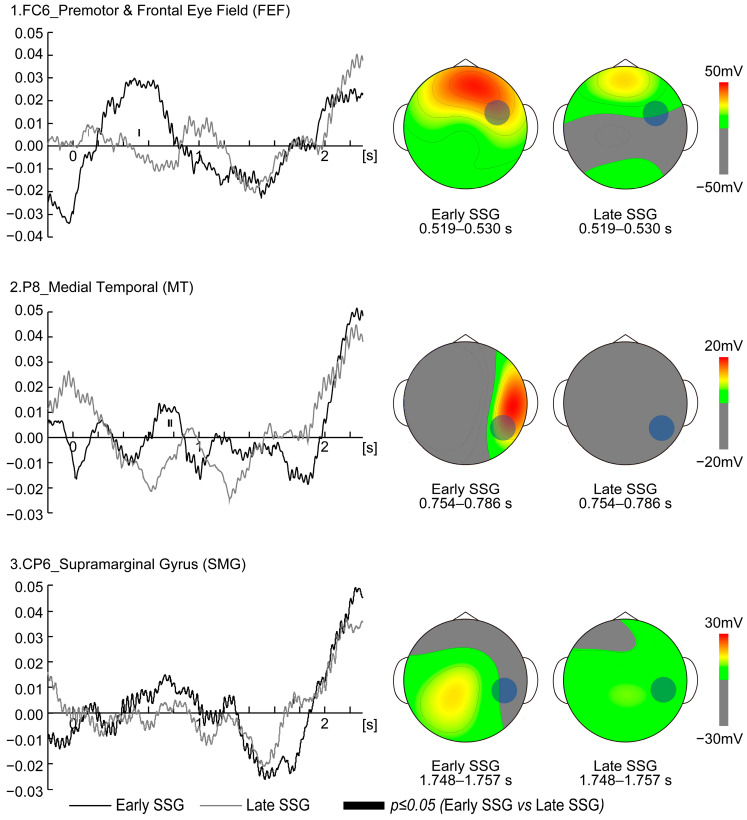
Electroencephalographic comparison between Early and Late Saccade Strategy Groups. Grand-averaged EEG signals in the low beta band (13–15 Hz) were extracted using the Hilbert transform during the 2.3 s visible period for the Early and Late Saccade Strategy Groups (SSGs). The left panels display average time-series EEG data from electrodes showing significant differences between the two groups; black lines represent the Early SSG, while gray lines represent the Late SSG. Time (relative to target onset) is shown on the *X*-axis, and significant group differences (*p* ≤ 0.05, non-parametric paired permutation test) are indicated by black bars at the bottom of the graphs. The right panels show scalp topographies of low beta activity for each group, averaged over the time intervals that exhibited significant differences at the FC6, P8, and CP6 electrode sites.

Figure 3 displays time-series EEG data for electrodes that showed significant group differences. The permutation analysis revealed significantly greater low beta activity in the Early SSG compared to the Late SSG at electrode FC6 between 0.519 and 0.530 s (*p* ≤ 0.05) and at electrode P8 between 0.754 and 0.767 s as well as 0.776 and 0.786 s (*p* ≤ 0.05) following target onset (Figure 3, left panel). Conversely, the Late SSG exhibited significantly greater low beta activity at electrode CP6 between 1.748 and 1.757 s (*p* ≤ 0.05) (Figure 3, left panel).

The spatial distribution of these significant differences across electrodes is depicted in the topographical representation in Figure 3 (right panel). The topographic map for P8 was created based on the time range from 0.754 to 0.786 s after target onset, which included two consecutive periods of statistical significance (0.754–0.767 s and 0.776–0.786 s).

## 4. Discussion

Electrode FC6 is located in Brodmann Areas 6 and 8 (BA6, BA8) based on Talairach coordinates (*x* = 60.5 ± 2.8 mm, *y* = 4.9 ± 7.3 mm, *z* = 25.5 ± 7.8 mm) [20]. This region overlaps with the premotor cortex (BA6) and the frontal eye field (BA8), both of which are critical for saccade generation and visuomotor transformation, as demonstrated in previous cerebral blood flow and lesion studies [21]. In the present study, low beta activity at FC6 was significantly higher in the Early SSG than in the Late SSG between 0.519 and 0.530 s after target movement onset, suggesting enhanced visuomotor transformation during this period [22]. Notably, the latency from FEF stimulation to saccade onset is approximately 30–45 ms [23]. Based on this latency, the increased FC6 activity observed at 0.519–0.530 s could correspond to saccades occurring around 0.550–0.575 s post target onset. Eye-tracking data showed that the gaze reached approximately 500 pixels 30–45 ms after the FC6 activity peak, supporting the notion that FC6 activation in the Early SSG facilitated rapid transformation of visual input into predictive saccade strategies. In contrast, the Late SSG showed suppressed FC6 activity, implying a reliance on real-time tracking rather than predictive saccades. These findings suggest that FC6 activity is temporally aligned with visuomotor transformation processes and highlights strategic differences in gaze behavior between the Early and Late SSGs.

At electrode P8, significantly greater low beta activity was observed in the Early SSG compared to the Late SSG at 0.754–0.767 s and 0.776–0.786 s after target onset. P8 corresponds to BA19 in the right hemisphere (*x* = 56.4 ± 3.7 mm, *y* = −64.4 ± 5.6 mm, *z* = 0.1 ± 8.5 mm based on Talairach coordinates) [20], located in the MT visual area, which is crucial for processing the speed and direction of moving stimuli [23]. This timing suggests that participants in the Early SSG had already begun shifting their gaze toward the predicted target endpoint, whereas those in the Late SSG continued to track the moving target. Although vergence was not measured in this study, it is plausible that Early SSG participants adjusted fixation depth, possibly using peripheral vision for final trajectory adjustments. This strategy is reminiscent of visual behavior observed in baseball outfielders, who estimate the trajectory early and then use peripheral cues for refinement. Importantly, the MT area recovers quickly from saccade-induced visual suppression, supporting stable motion perception post saccade [24]. The elevated P8 activity in Early SSG participants likely reflects greater reliance on predictive processing and motion cues to anticipate the target’s final position. In summary, the MT area appears to facilitate post-saccadic motion perception and supports predictive gaze strategies. The differential P8 activity between groups underscores contrasting visual strategies, with the Early SSG leveraging peripheral vision and anticipatory mechanisms to enhance accuracy.

Electrode CP6 is located in Brodmann Areas 40 (BA40) based on Talairach coordinates (*x* = 62.9 ± 3.7 mm, *y* = −44.6 ± 6.8 mm, *z* = 24.4 ± 8.4 mm), and it lies within in the supramarginal gyrus (SMG) [20]. This region is implicated in various cognitive and sensorimotor processes, including the interruption of a current goal to reorient attention toward a salient stimulus [25,26]. Therefore, CP6 activation for the Late SSG may reflect increased demands for visual target selection and gaze adjustment. Unlike the FEF or the superior colliculus (SC), which are directly responsible for saccade generation, the SMG is more likely involved in spatial information processing and the modulation of eye movements in response to unexpected changes [25,26]. This suggests that for the Late SSG, CP6 activity represents a compensatory mechanism for delayed saccadic adjustments, rather than direct involvement in saccade initiation. At 1.748–1.757 s after target onset, participants in the Late SSG were still actively tracking the moving target, whereas those in the Early SSG had already shifted their gaze to the predicted arrival position. The elevated CP6 activity in the Late SSG indicates continued visual information extraction at this time point, likely to refine predictive estimates. This delayed engagement of the SMG suggests a reliance on visually guided adjustments rather than anticipatory strategies. Furthermore, the observed delay in gaze shift—occurring approximately one second after the CP6 activity peak—supports the idea that Late SSG participants were still processing visual input rather than executing a predictive saccade. Given that MT activity in the Early SSG and SMG activity in the Late SSG are associated with visual processing, the timing of motion detection appears to be a critical factor in determining the saccade strategy. Participants who can detect and process motion patterns early tend to adopt an Early SSG strategy, while those relying on prolonged visual sampling exhibit higher CP6 activation and favor the Late SSG approach.

These EEG findings suggest that early predictive saccade strategies are associated with enhanced activation in visuomotor and motion-sensitive regions. Although the final behavioral outcomes (e.g., position and response errors) did not differ significantly between groups, the early divergence in eye movement strategies—particularly in gaze timing and trajectory—was accompanied by distinct neural activity patterns. This supports the view that EEG measures may capture underlying processing differences not always reflected in overt task performance. We therefore interpret these neural differences as complementary to behavioral measures, offering a more nuanced perspective on visuomotor prediction strategies.

However, it is important to note that these findings are correlational in nature and do not imply causal relationships between neural activation and strategy selection. Moreover, we acknowledge that other factors such as attentional engagement, task motivation, or fatigue may have contributed to the observed group differences in EEG activity. Additionally, the current study did not directly assess the potential use of peripheral vision during the predictive saccade task. While it is plausible that participants in the Early SSG may have relied on peripheral cues to anticipate the target’s landing point, future studies should investigate this possibility using experimental designs that explicitly manipulate peripheral visual availability.

This study focused on a specific task condition and involved a relatively small sample size, which may limit the generalizability of the findings. Future research with larger and more diverse samples is warranted. In particular, the Early SSG included only six participants, which may reduce the statistical power and robustness of group-level comparisons. Nevertheless, the observed consistency in both behavioral and EEG measures across participants provides preliminary support for the proposed classification. Replication with larger samples will be essential to confirm and extend these findings.

## 5. Conclusions

This study examined the neural dynamics underlying the timing of predictive saccade strategies under occlusion conditions. By classifying participants into Early and Late Saccade Strategy Groups based on their gaze behavior, we found that early saccade execution was associated with increased EEG activity in regions such as the FEF (FC6) and the motion-sensitive MT area (P8). These findings suggest that early initiation of predictive saccades more effectively engages visuomotor transformation and motion-processing areas. In contrast, delayed saccade strategies were linked to increased activation in regions associated with attentional reorientation (CP6), reflecting a more reactive, visually guided control strategy. Together, these results enhance our understanding of how the timing of visual prediction influences neural processing and provide practical implications for improving performance in traditional sports and eSports contexts.

## Figures and Tables

**Table 1:** Launch angles and velocities for each target type.

Target Type	Initial Velocity (°/s)	Launch Angle (°)	Highest Point (*x*, *y*) (cm)	Final Position (*x*, *y*) (cm)
CLOSE	23.28	75.96	(12.98, 26.00)	(25.97, 0.12)
MID	23.33	75.45	(13.48, 26.00)	(26.97, 0.12)
DISTANT	23.39	74.93	(13.98, 26.00)	(27.97, 0.12)

## Data Availability

The datasets generated during and/or analyzed during the current study are available from the corresponding author upon reasonable request due to ethical reasons.

## Abbreviations

The following abbreviations are used in this manuscript:

BA	Brodmann area
EEG	Electroencephalography
ERP	Event-related potential
FDR	False discovery rate
FEF	Frontal eye field
FPS	First-person shooter
MT	Middle temporal
PE	Position error
SC	Superior colliculus
SMG	Supramarginal gyrus
SSG	Saccade strategy group
